# The quantification of gesture–speech synchrony: A tutorial and validation of multimodal data acquisition using device-based and video-based motion tracking

**DOI:** 10.3758/s13428-019-01271-9

**Published:** 2019-10-28

**Authors:** Wim Pouw, James P. Trujillo, James A. Dixon

**Affiliations:** 1 Center for the Ecological Study of Perception and Action, University of Connecticut, Storrs, CT USA; 2 Department of Psychology, Education, & Child Studies, Erasmus University Rotterdam, Rotterdam, The Netherlands; 3 Donders Institute for Brain, Cognition, and Behaviour, Radboud University, Nijmegen, The Netherlands; 4 Centre for Language Studies, Radboud University, Nijmegen, The Netherlands

**Keywords:** Motion tracking, Video recording, Deep learning, Gesture and speech analysis, Multimodal language

## Abstract

There is increasing evidence that hand gestures and speech synchronize their activity on multiple dimensions and timescales. For example, gesture’s kinematic peaks (e.g., maximum speed) are coupled with prosodic markers in speech. Such coupling operates on very short timescales at the level of syllables (200 ms), and therefore requires high-resolution measurement of gesture kinematics and speech acoustics. High-resolution speech analysis is common for gesture studies, given that field’s classic ties with (psycho)linguistics. However, the field has lagged behind in the objective study of gesture kinematics (e.g., as compared to research on instrumental action). Often kinematic peaks in gesture are measured by eye, where a “moment of maximum effort” is determined by several raters. In the present article, we provide a tutorial on more efficient methods to quantify the temporal properties of gesture kinematics, in which we focus on common challenges and possible solutions that come with the complexities of studying multimodal language. We further introduce and compare, using an actual gesture dataset (392 gesture events), the performance of two video-based motion-tracking methods (deep learning vs. pixel change) against a high-performance wired motion-tracking system (Polhemus Liberty). We show that the videography methods perform well in the temporal estimation of kinematic peaks, and thus provide a cheap alternative to expensive motion-tracking systems. We hope that the present article incites gesture researchers to embark on the widespread objective study of gesture kinematics and their relation to speech.

There is an increasing interest in the ways that co-occurring hand gestures and speech coordinate their activity (Esteve-Gibert & Guellaï, 2018; Wagner, Malisz, & Kopp, 2014). The nature of this coordination is exquisitely complex, as it operates on multiple levels and timescales. For example, on the semantic level, referential gestures can augment (or highlight) speech by iconically denoting state of affairs that are *not* expressed in speech. Twirling the finger when saying “they went down the stairs” can thereby indicate that the stair was a spiral staircase (McNeill & Duncan, 2000). Twirl the fingers a couple of seconds too early, and the multimodal message becomes ambiguous. Therefore, timing matters. Timing is even more finely tuned on the prosodic level, where it has been found that gesture–speech coupling occurs on even shorter timescales (200 ms). For example, it has been found, in the pronunciation of the nonsense word “baba,” when stress is put on the first (***ba***ba) or last syllable (ba***ba***), the pointing gesture’s maximum extension is coordinated to align more closely with the stressed syllable (Rochet-Capellan, Laboissiere, Galvan, & Schwartz, 2008; see also Esteve-Gibert & Prieto, 2013; Krivokapić, Tiede, & Tyrone, 2017; Rusiewicz, Shaiman, Iverson, & Szuminsky, 2014). On the biomechanical level, it has further been found that gesture’s moments of peak physical impetus (lasting ~ 50 ms) entrain fundamental frequency and the amplitude envelope during phonation (Pouw, Harrison, & Dixon, 2019). Thus, the gesture–speech relationship is defined over many levels and timescales (for overviews, see Kendon, 2004; Wagner et al., 2014).

The study of the temporal dynamics of gesture–speech coordination has relatively lagged behind in use of *kinematic measurement methods*, especially as compared to the degree to which state-of-the-art (psycho)linguistic methods are employed for the study of speech (e.g., Loehr, 2012; Shattuck-Hufnagel & Ren, 2018). This manifests itself in the relative scarcity (as compared to other research on instrumental action) of published studies that have applied motion tracking in *gesture–speech* research (Alexanderson, House, & Beskow, 2013; Alviar, Dale, & Galati, 2019; Chu & Hagoort, 2014; Danner, Barbosa, & Goldstein, 2018; Ishi, Ishiguro, & Hagita, 2014; Leonard & Cummins, 2010; Krivokapic, Tiede, & Tyrone, 2017; Krivokapić, Tiede, Tyrone, & Goldenberg, 2016; Parrell, Goldstein, Lee, & Byrd, 2014; Pouw & Dixon, 2019a; Quek et al., 2002; Rochet-Capellan et al., 2008; Rusiewicz et al., 2014; Treffner & Peter, 2002; Zelic, Kim, & Davis, 2015). It can be argued that the absence of motion tracking in the standard methodological toolkit of the multimodal language researcher has further led to imprecisions and conceptual confusions. For example, in the quantification of how tightly gestures couple with prosodic markers (e.g., pitch accent) in speech, researchers have pinpointed relevant kinematic events in gesture by manually identifying the point of “maximum effort” from video recordings (Wagner et al., 2014). Others have used further clarifying definitions of Adam Kendon (2004), which suggests that the researchers should search for the “kinetic goal” or the “peak of the peak” of the gesture (see Loehr, 2004, p. 77). Although, such kinematic judgments are generally made by several raters allowing for some measure of objectivity, the resolution of the kinematics of gesture is necessarily constrained when working with nonquantitative definitions. Wagner highlights that non-quantitative definitions have led to conceptual confusions that have made the literature markedly difficult to digest:

[the maximum effort is studied] with varying degrees of measurement objectivity and with varying definitions of what counts as an observation of maximum effort. Most definitions evoke a kinesthetic quality of effort or peak effort . . . correlated with abrupt changes in visible movement either as periods of movement acceleration or strokes . . . , as sudden halts or hits . . . , or as maximal movement extensions in space called apexes. . . . (Wagner et al., 2014, p. 221; original emphasis, citations removed)

Thus, as highlighted above, conceptual confusions can result from using nonquantitative definitions. The further positive implication of using quantitative methods of gesture–speech coordination is that new structured behaviors can be uncovered with multimodal tracking techniques that are otherwise simply hidden from view. For example, Alviar et al. (2019) have recently found that lecturing speakers organize their speech and movement in relation to the changing environment (e.g., changing slides) as a single system, in a way that allows for statistical dimensional reduction of its overall activity. For their analyses, it was essential to have continuous synchronized data of speech; such data are impossible or otherwise impractical to acquire without quantitative methods. This was equally the case for a study by Parrel and colleagues (Parrell, Goldstein, Lee, & Byrd, 2014) who showed that in a finger-tapping and speaking task the coupling of gesture and speech leads to subtle *unintended* changes in one modality because of *intended* changes in the other modality. In an effort to understand the degree to which gesture and speech are bidirectionally coupled as opposed independently operating, McNeill (1992) in a classic study had participants retell a cartoon while hearing their speech on a delay of about 200 ms. This delay is known to slur and hamper speech fluency. At the time, McNeill could only infer from video data that gesture and speech still seemed to synchronize properly. Recent motion-tracking research has not only been able to confirm McNeill’s initial observations (Chu & Hagoort, 2014; Rusiewicz et al., 2014), but it has uncovered that gesture and speech in fact become more synchronized under delayed auditory feedback and that gesture is slightly entraining to the delayed feedback signal (Pouw & Dixon, 2019a). These findings helped to expand a dynamical systems account of gesture–speech synchrony that could not have been tested without high-resolution data streams because said effects operate on timescales of about 200 ms (Pouw & Dixon, 2019a).

Although the previous examples show that multimodal quantitative data can uncover hidden structure in basic speech-gesture synchrony, such data can also be informative in clinical research. For example, whereas early research in autism showed impaired gesture use, more recent work using manual annotation has shown that autism may be characterized by atypical synchrony between speech and gesture (de Marchena & Eigsti, 2010). Applying quantitative methods could allow a better understanding of this phenomenon, and thus also of communicative behavior in autism. There are many more examples (e.g., Rochet-Capellan et al., 2008; Treffner & Peter, 2002; Zelic et al., 2015), but the point that we want to make is that given that the timescales at which speech and gesture couple are not easily accommodated by classic methods in psychology, more widespread adoption of quantified multimodal tracking will likely lead to uncovering structure that is otherwise hidden. Such quantitative multimodal tracking additionally opens the door to extracting more complex features from the kinematics of gestures (e.g., Mittelberg, 2018; Trujillo, Vaitonyte, Simanova, & Özyürek, 2019; Pouw & Dixon 2019b), allowing research into the dynamics of speech and gesture at a more abstract level.

Finally, mainstream hand-coded methods for identifying properties of gesture kinematics are notoriously time-consuming, but notably are still being proposed (Hilliard & Cook, 2017), given the absence of viable and easy-to-implement alternatives. As Wagner et al. (2014) and Danner (2017) also acknowledge, the time-consuming aspect of multimodal research has had the implication that studies on gesture–speech synchrony are typically performed with single or a limited number of subjects that are intensively studied with a micro-level approach (e.g., Leonard & Cummins, 2010; Loehr, 2012; Shattuck-Hufnagel & Ren, 2018). Thus, the time intensiveness of the current methodology limits the amount of data that can be generated, which then serves to weaken generalizability of effects to the larger population and the role of individual differences therein.

It is clear, therefore, that if the laborious task of hand coding gesture kinematics can be replaced with reliable and objective automated methods, considerable advantages will accrue for researchers. In the present article, we contribute to this joint effort by introducing and validating novel methods for studying gesture–speech dynamics (e.g., Beecks et al., 2015; Danner et al., 2018; Krivokapić et al., 2017; Schueller et al., 2017; Pouw & Dixon 2019b), and by providing a tutorial for overcoming some common challenges when doing multimodal research.

## Overview

In Part I, we address some key steps that need to be taken in order to study multimodal communication in a quantified and replicable manner. In the first section, we focus on introducing relevant motion-tracking methods. Then we specifically focus on the multimodal data processing of gesture in relation to speech. In the final section of Part I, in tutorial fashion, we provide solutions and R code for data-recording and data-processing challenges.

In Part II of this article, we provide a quantitative validation of two inexpensive motion-tracking videography methods (a pixel differentiation and a deep neural network approach) by comparing performance of these approaches with a high-performance standard: a wired motion-tracking system called the Polhemus Liberty. By validating that videography methods are suitable for use in multimodal research, we hope that we can further fuel the widespread kinematic study of gesture.

## Part I: Key steps to quantifying speech–gesture synchrony

First, we discuss the major motion-tracking approaches currently available, followed by a brief tutorial on synchronizing audio-visual data streams, and concluding with a suggestion for how to annotate and assess the combined data.

### Motion-tracking methods

The first challenge for the multimodal language researcher is to decide which type of motion-tracking system is needed to answer a particular research question. In this section, we provide an overview of the principal motion-tracking methods currently available. Table 1 provides a summary overview of these methods, as well as suggestions for their application.

**Table 1:** Overview of motion-tracking methods

Method	Key features	Cost level	Application
Video-based			
Pixel differentiation	Simple to compute; Requires very stable background	Low	Calculation of overall movement and velocity in relatively constrained data
Computer vision	Can track very specific parts of the scene (e.g., hands, face); Computationally costly	Low	Tracking specific body parts and/or movements of multiple people
Device-based			
Wired	High precision and robust against occlusion; Limited by number of wired sensors that can easily be attached	High	Focus on a small number of articulators, for which precision is needed and occlusion may be a problem for other methods
Optical (markered)	Gold-standard precision; Requires calibration and for participants to wear visible markers	High	High precision tracking of multiple body parts on one or multiple participants
Markerless (single-camera)	Non-invasive, 3-D tracking; Lower precision and tracking stability	Moderate	Mobile setup for whole-body tracking when fine-grained precision is less necessary

Motion-tracking methods can be broadly separated into two categories: video-based tracking, which utilizes standard video recordings to measure movements, and device-based tracking, which requires specialized hardware to measure movements. Note that we do not wish to give an exhaustive overview of all motion-tracking methods, but rather provide an introduction to some of the more widely known or commonly employed methods. The aim is to provide the reader with an overview of the common approaches to motion tracking, as well as to introduce some specific implementations of these methods.

#### Video-based tracking

Video-based tracking has a long history in the field of computer vision, and therefore many approaches are available. This approach is an attractive option because it can be applied to video data that has already been acquired. As such, it can be a powerful tool for multimodal language researchers who sometimes work with large corpora of preexisting video data. This approach is also highly accessible, as much of the software is freely available. Although video-based tracking can provide an accessible and useful measure of movement, it is limited by the field of view of the original recording, as well as by the fact that 3-D movement must be estimated from 2-D data.

Perhaps the simplest approach to estimating movements is pixel-based motion tracking. This type of tracking typically takes advantage of a process known as “optical flow.” In this approach, movement is quantified on the basis of the change of pixels from one frame to the next. Starting with a two-dimensional vector field representing all pixels in the scene, the rate and location of changes in the brightness of these pixels lead to the calculation of speed and direction of movement within the scene.

Overall, pixel differentiation has been shown to provide a reliable measure of movement (Paxton & Dale, 2013; Romero et al., 2017) and can be used to capture movement in specific areas of the visual scene (Alviar et al., 2019; Danner et al., 2018). Note, however, that this method is particularly vulnerable to changes in background, such as movement or changes in lighting, and may not be able to capture smaller movements or movements toward the camera. Furthermore, motion tracking of multiple individuals is challenging if individuals move in a close proximity, as the bodily regions of interest that are tracked are likely to overlap, leading to inaccuracies in movement estimates of the individuals. In sum, if dealing with suitable quality video data and movements that are likely to be well tracked by this approach, pixel differentiation can provide easily accessible and robust measure of continuous movement.

Novel methods that will likely revolutionize the study of movement with video data utilize deep learning methods, like OpenPose. OpenPose was developed by Cao, Simon, and Wei (2017) as a method of computer-vision based estimation of bodies from 2-D still frames or videos. The method uses a form of deep learning (see LeCun, Bengio, & Hinton, 2015, for a review of this topic), specifically convolutional neural networks (LeCun & Bengio, 1995), to predict the location of body parts as well as to ensure that body parts are consistently assigned to the correct individual. OpenPose offers an advantage over more simplistic pixel-based methods of tracking, because it allows the simultaneous tracking of multiple individuals present in a scene. Since the neural network is trained to detect specific body parts, it is also more robust to background noise and images involving multiple people moving and interacting at once.

The currently available version of OpenPose provides multiperson tracking of the body, face, and hands. The library uses predefined key points (e.g., shoulder, elbow, wrist), with the number and exact location of the key points varying slightly depending on the library used. The method, therefore, provides an excellent solution for estimating movement of the body or hands in 2-D, with an off-the-shelf (i.e., pre-trained) network ready to use for estimation of standard points of interest on the body.

Similar to OpenPose, Deeplabcut (Mathis et al., 2018) uses deep learning to estimate movement from video data. DeepLabCut is a derivative of an earlier approach, called DeeperCut (Insafutdinov, Pishchulin, Andres, Andriluka, & Schiele, 2016), which estimated whole-body poses (i.e., body part positions) from video data. Although to date no direct comparisons have been made between DeeperCut and OpenPose, both have independently shown excellent performance in detecting human poses in video and image data. DeepLabCut utilizes the feature detectors from DeeperCut and retrains these detectors on new inputs. The feature detectors are the readout layers from DeeperCut that provide the predicted location of a body part. By training these feature detectors on new inputs, DeepLabCut effectively “rewires” the network, creating feature detectors for a new input. This approach is appealing because this rewiring of the network allows the researcher to define which objects or body parts should be detected, granting a large amount of flexibility to the researcher.

One of the main advantages to DeepLabCut is that its use of a pre-trained network and an “extremely deep neural network” (Mathis et al., 2018) leads to a relatively small amount of data required to train the model. Whereas most pose estimation models, such as DeeperCut, require thousands of labeled images for training, DeepLabCut achieves high performance with only ~ 200 labeled images. Although the model training requires more work from the researcher than off-the-shelf pose estimators like OpenPose, it provides a powerful tool for researchers who wish to define their own points of interest to track.

#### Device-based tracking

Although video-based tracking methods are useful for extracting movement information from data that is already acquired, such as video corpora, the gold standard for capturing human movement remains device-based tracking. This is primarily due to the generally higher *spatiotemporal resolution* of device-based recordings. They can sample body position more often, and they provide more accurate spatial estimates of body position. Furthermore, device-based tracking captures movement three dimensions, and is thus not confined to the vertical and horizontal planes, as is the case with videography methods.

Next we focus on three specific subtypes of device-based tracking: wired motion tracking, optical motion tracking, and markerless motion tracking. We provide a specific example of each subtype, selected on the basis of what we believe are the most widely used.

##### Device-based wired motion tracking

Currently, the Polhemus Liberty (Vermont, USA; http://www.polhemus.com; Liberty Latus Brochure, 2012) is one of the most widely used devices for motion-tracking in the study of human action and gesture (e.g., Pouw & Dixon, 2019a; Treffner & Peter, 2002). The Polhemus system utilizes a set of electromagnetic sensors that can be attached to the body, with movement captured by a set of receptors that together define the recording space. With a temporal resolution of 240 Hz (samples per second) per sensor, a resolution 0.0012 mm for ideal conditions (30-cm range; total possible range is 180 cm), the system provides a fine-grained capture of movement. Because the recordings are based on electromagnetic fields, the system also does not suffer from occlusions, so even complex movements are captured robustly.

Although the Polhemus Liberty is a powerful tracking tool, researchers may be limited in how much overall body movement can be captured due to the cost and setup of many wired sensors. Additionally, due to its reliance on an electromagnetic field, the system cannot be used in conjunction with other electronic methods such as EEG, and care should be taken to keep metals away from the system’s tracking range. Careful pretesting of the motion-capture area is recommended, so that distortions from electromagnetic fields do not affect data collection. Wired motion tracking is therefore especially useful for measuring gestures in specific body parts, which may not be confined to two dimensions of movement, and for analyses requiring a fine-grained resolution of movement.

##### Device-based optic marker motion tracking

Optic tracking typically uses infrared light that is captured by several cameras, with the 3-D position of a physical marker being calculated on the basis of the multiple viewpoints of the camera array. One type of system uses wired, infrared light-emitting diodes that are placed on key points on the body. A popular example of this type of system is the Optotrak system (Northern Digital, Waterloo, Canada). The Optotrak is known for high reliability (States & Pappas, 2006), high temporal resolution (max 400 Hz), and high spatial resolution (approximately 0.1 mm at 2.25 m distance from the cameras). Although Optotrak’s motion tracking is of high quality, practical implementation may require some care and expertise. For example, States and Pappas discussed the problem that tracking quality deteriorates when the markers are tilted away from the plane of the sensors.

Another type of optic system does not use wired sensors, but instead requires participants to wear reflective markers that are subsequently tracked by an array of cameras that emit infrared light. Similar to wired optical motion tracking, nonwired optical tracking is known for its high precision, with the Vicon system providing a temporal resolution of 100 Hz as well as submillimeter spatial resolution. The Vicon system, in particular, has consistently been shown to be one of the most reliable and precise motion tracking systems (Richards, 1999; Vigliensoni & Wanderley, 2012), even exceeding the Polhemus Liberty (Vigliensoni & Wanderley, 2012).

Although both wired and nonwired optical tracking provide high precision, they also require calibration and somewhat intrusive markers to be placed on participants body. Given that participants need to wear markers, these systems maybe not be ideal when working with children or sensitive populations. For example, it has previously been noted that sensors or markers that must be attached to the body can be stressful or distracting to children with autism (Anzulewicz et al., 2016; Romero et al.,^2018^). Researchers opting for marker-based tracking should therefore take care to ensure that their study population is not overly sensitive to tactile stimulation (e.g., autistic children), potentially “unnatural” feeling test environments, or the distraction of physical markers being attached to their body (e.g., very young children). Additionally, although precision may be higher for Vicon than Polhemus, the Vicon system is more prone to tracking loss due to occlusion of the reflective markers. Researchers considering these methods should therefore consider the types of movements that participants may produce, and how they may respond to the physical attachment of markers.

##### Device-based markerless motion tracking

A somewhat new addition to motion tracking technology is the use of markerless devices. One such device is the Leap Motion, which uses three infrared emitters and two infrared cameras. Measuring the deformation of the (reflected) infrared light allows the Leap Motion to capture 3-D shapes in its field of view. The device has a high spatial (0.4–1.2 mm) and a reasonable temporal (mean = 40 Hz) resolution, and has been shown to be quite reliable in its spatial tracking (Weichert, Bachmann, Rudak, & Fisseler, 2013). The primary limitations of this device are its relatively small field of view (Guna, Jakus, Pogačnik, Tomažič, & Sodnik, 2014), which requires gestures to be produced directly above the device, and its inconsistent sampling rate (Guna et al., 2014). Its high spatial resolution makes the Leap Motion ideal for experiments measuring fine-grained hand and finger movement in a confined area in space. However, it is less ideal for capturing gestures in a less constrained environment or for larger movements involving the limbs or other body parts.

Similar in concept to the Leap Motion, the Microsoft Kinect uses a combination of infrared depth cameras with computer vision algorithms, allowing markerless estimation of key points on the whole body using 3-D tracking. The primary advantage of the Kinect is that it provides unobtrusive 3-D motion tracking of the major articulators, such as head and gross arm/hand movements. The system is also relatively low cost and very mobile, allowing one to capture data outside of a confined lab setting. Open-source Kinect recording software is also available, such as OpenKinect (10.5281/zenodo.50641), making the system a highly accessible motion-tracking device. Kinect-based tracking has been shown to be reliable for several tasks when compared to gold-standard systems of assessment. For example, Otte et al. (2016) show excellent agreement between the Kinect and Vicon systems for clinical assessments of motor function. Additionally, Kinect-based measures of gesture kinematics have also been validated against human coder assessments of the same features (Trujillo et al., 2019).

With a temporal resolution of 30 Hz, the Kinect does not offer the fine-grained resolution of markered or wired tracking systems. Although the Kinect can provide reliable tracking of larger articulators, such as arm and hand gestures, there is also evidence that Kinect is much less precise in capturing fine-grained movements, such as finger tapping (Romero et al., 2017), when compared to systems such as Polhemus or Vicon (Vigliensoni & Wanderley, 2012). The mobility and nonintrusive nature of Kinect must therefore be carefully weighed against its reduced precision.

### Multimodal data acquisition: Synchronization of audio–visual motion-recording streams and multimodal annotations

Having decided on what type of motion-tracking method is applicable to your research question, the next hurdle is to have the multimodal data streams synchronized in their recording. Three streams of data are most relevant for multimodal language research: audio, video, and motion tracking. Motion trackers are often stand-alone devices, without in-built audio recording capabilities. If audio recording is provided (such as Kinect) the audio quality is often subpar for acoustic analyses. Thus, in most cases motion tracking needs to be synchronized in some way with the audio and the video stream. To complicate matters further, although generic video cameras do record audio (and thus have audio and visual streams synchronized by default), the quality of the audio recording is often surpassed by more specialized audio recording equipment. Furthermore, specialized stand-alone audio equipment is often preferable as this equipment can be tailored for high performance recording in specific situations; for example, in noisy environments, one can filter surrounding noises using condenser cardioid microphones. Thus, if one prefers high-grade tracking of motion, audio, and video, one is often confronted with having to synchronize data streams recorded from three different pieces of equipment.^1^

If built-in synchronization of video is not possible, how do we synchronize the separate recording of audio and movement? A possible solution is activating the recording of the audio stream and the motion-tracking (e.g., Polhemus) stream via a single personal computer (PC) system. For the dataset that we use in Part II, we handled near-simultaneous activation (within a few milliseconds; also dependent on system specifications) of the recording of Polhemus motion-tracking data and the microphone using a C++ script made openly available by Michael Richardson (2009) that we further modified for audio recording using SFML audio packages (toolbox SFML for C++; for a link to this modified script for audio motion tracking with a Polhemus Liberty system, see the folder “c++ code Polhemus + Audio” at https://osf.io/rgfv3/).

Although we use this particular solution, there are many methods for synchronizing audio and motion tracking (and video). Most notable in this case is “Lab Streaming Layer” (Kothe, 2014; for a demo, see https://www.youtube.com/watch?v=Y1at7yrcFW0&t=539s), which provides a way to synchronize recordings from multiple PC or laptop systems at once. The program can be further customized and implemented using the Python or C++ programming languages. Lab Streaming Layer is particularly suitable when, for example, a simultaneous recording is needed with more than one microphone or two motion-tracking systems. Indeed, recording from two microphones often requires a specialized device (e.g., PC with two sound cards installed), or otherwise requires two separate devices that need to be synchronized in their recording as well (next to video- and motion-tracking data). Lab Streaming Layer in this case is ideal, as it can coordinate recording of multiple devices from multiple systems, which makes it an excellent solution for dyadic or multiperson language research. However, we note that these solutions require some programming skills; they are not run by graphical user interfaces. Audio and video recording from multiple systems can also be synchronized post-hoc in an easy way as we will introduce below.

If the motion tracking and the audio are synchronized in their recording onset and offset, such as in our case, the video recording still needs to be synchronized in the post-processing phase with the audio and motion data. This synchronization is necessary in most cases, because in the annotation phase (using ELAN) we want to have both the motion-tracking data and high-grade audio aligned with our video data so as to be able to make accurate decisions about gesture–speech typology. For example, ELAN allows one to import movement time-series data into the time line (Crasborn, Sloetjes, Auer, & Wittenburg, 2006), such that gesture initiation can be aligned with motion tracking output (see the discussion below on gesture annotation).

We obtained an easy solution for post-hoc audio synchronization using Adobe Premiere Pro CC 2015.^2^ Adobe Premiere Pro CC allows the user to load in multiple audiovisual or multiple audio-only streams, and then apply the function “synchronize audio” for these streams. The synchronization of the audio is performed by Adobe Premiere Pro by aligning the temporal structure of waveform *A* (e.g., in-build camera audio) with the temporal structure of waveform *B* (e.g., microphone audio). Given that in our case the camera and the microphone have recorded a single (or at least partially shared) audio event (speech of the participant), the alignment of the waveforms is possible. By aligning the waveforms of the (a) camera audio and the (b) microphone, coincidentally, the (c) video and (d) motion-tracking + audio data are also aligned (given that **a** was already synchronized with **c**, and **b** was already synchronized with **d**). Using this *chaining technique* of synchronization allows one to synchronize a host of devices and data streams, as long as each system has a “mother” audio stream that can link to the other systems’ “mother” audio streams.^3^

#### Creating multimodal annotations

Once the experiment is completed and raw data are recorded, the researcher often still needs to isolate some relevant events from the data based on expert judgment. For example, prosodic markers may need to be applied using the ToBi method (Beckman & Elam, 1997), which requires several expert raters to judge different aspects of speech (e.g., when pitch accents were unfolding; cf. Loehr, 2012; Shattuck-Hufnagel & Ren, 2018). Similarly, gesture events may need to be identified, which will always involve some hand-coding of particular gesture types (e.g., beat vs. iconic gestures).

ELAN is a well-known, powerful research tool that can be used during the annotation phase (Lausberg & Sloetjes, 2009). We will not go into how to use ELAN, but we do want to highlight two important ELAN functionalities that are particularly helpful when doing motion-tracking research. First, the time-series data from the motion tracker can be uploaded to ELAN, which allows you to continuously monitor movement trajectories as a visual aid during the annotation of bodily gestures (see Crasborn et al., 2006; for an example from Pouw & Dixon, 2019a, see https://osf.io/5h3bx/). This visual aid is particularly superior to the raw video for deciding *when* a gesture initiates and ends, because of the minimalist representation of movement and the sampling rate of the motion tracker, which is likely to yield higher visual and temporal resolution for the gesture coder.

More objective estimates for gesture-initiation and termination can be used when one has motion-tracking data, which can be employed within ELAN as well. One simple approach using raw motion capture data is the Elan Plugin for Automatic Annotation (EPAA). The EPAA allows the researcher to automate gesture detection by providing some arbitrary cut-off for when a particular movement reaches a speed or velocity threshold. For example, Hassemer (2016) used EPAA by applying a cutoff speed of the hands of greater than 10 cm/s to allow for a first automated pass for likely gesture events, which was then further modified by expert judgment. The motion-tracking approaches described above allow several alternative approaches, typically based on peaks in movement speed. For example, methods for video-based, semi-automatic gesture identification have recently been described. For example, Danner et al. (2018) employed pixel differentiation to identify gesture strokes based on peaks in movement found in a specified area of the video (see the Video-Based Tracking: Pixel Differentiation section for more detail on the exact implementation). De Beugher, Brône, and Goedemé (2014) introduced a custom hand-detection algorithm paired with a gesture recognition approach based on displacement from automatically calculated rest positions. Alternatively, Trujillo et al. (2019) described an approach using several kinematic features extracted from 3-D motion tracking, such as Kinect, to support gesture annotation.

### Basic tutorial of multimodal data-processing

After data collection, how does one process the different data streams so as to ready them for analysis? This is nontrivial as often one has a particular speech time series, such as fundamental frequency (F0), which still needs to be merged with the motion-tracking time series. A challenge that may arise in merging speech and motion-tracking data is that the sampling rate of the speech time series and the sampling rate of the motion tracker may be different. For example, videography motion tracking might sample at 29.97 Hz, whereas the F0 track of speech may sample at 240 Hz. This example would give us the file in Data Example 1.

**Data Example 1** Raw speech (SP_DATA) and motion-capture (MOC_DATA) time series

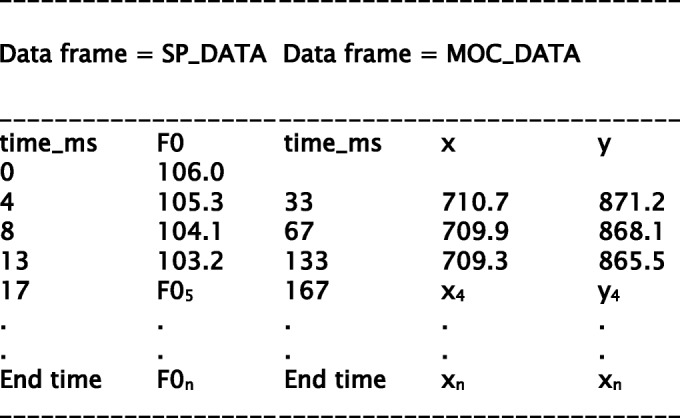



The left data frame, called SP_DATA, shows an example of the fundamental frequency of speech in hertz (i.e., pitch track), which samples at 240 Hz. The right data frame, called MOC_DATA, is an example of 2-D position motion capture data, which samples at 29.97 Hz, a sampling rate that is common for videography methods (about every 33.367 ms).

In this case, we not only need to align the datasets, but we also need to up-sample the motion-capture data (MOC_DATA to 240 Hz) or down-sample the speech data (SP_DATA to 29.97 Hz), if we want to end up with a fully merged speech + motion data file. Often we want to keep the high sampling rate of speech (in this case, 240 Hz), rather than reduce resolution of speech so as to merge with lower-sampling motion data (29.97 Hz). Thus we need to “up-sample” our motion-capture data.

First, to merge the motion-capture data with the speech data, the following base function from R, called “merge,” will align the datasets and merge them in a single data frame called “merged_file” (see R Code Example 1).
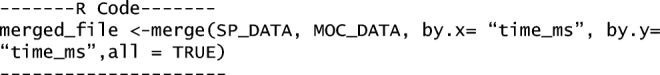


**R code Example 1** Merging two data frames

**--------R Code--------**

**merged_file < - merge(SP_DATA, MOC_DATA, by.x= “time_ms”, by.y = “time_ms”, all = TRUE)**

**-----------------**

This R code constructs a new data frame called “merged_file,” wherein the MOC_DATA time-series data will be merged with SP_DATA on the basis of the reference time variables “time_ms” present in both datasets. “All = TRUE” indicates that new rows will be created whenever “time_ms” from the speech data and “time_ms” from the MOC_DATA are not identical; for those rows, only data for one of the data streams are present (the other will have NAs).

Applying this function will give you the following file (Data Example 2), in which each observation from the speech and motion tracking datasets are now collected at some time *t* (or “time_ms”) in the merged dataset, and observations are merged together on one single row if possible (i.e., when at time *t* both F0 and motion-capture observations are made). For example, if at some time *t* (e.g., time_ms = 234 in Data Example 2) a motion-capture observation is present but no F0 observation, then a row will be constructed with only data for the motion-capture observation for that row.

**Data Example 2** Raw speech (SP_DATA) and motion-capture (MOC_DATA) time series

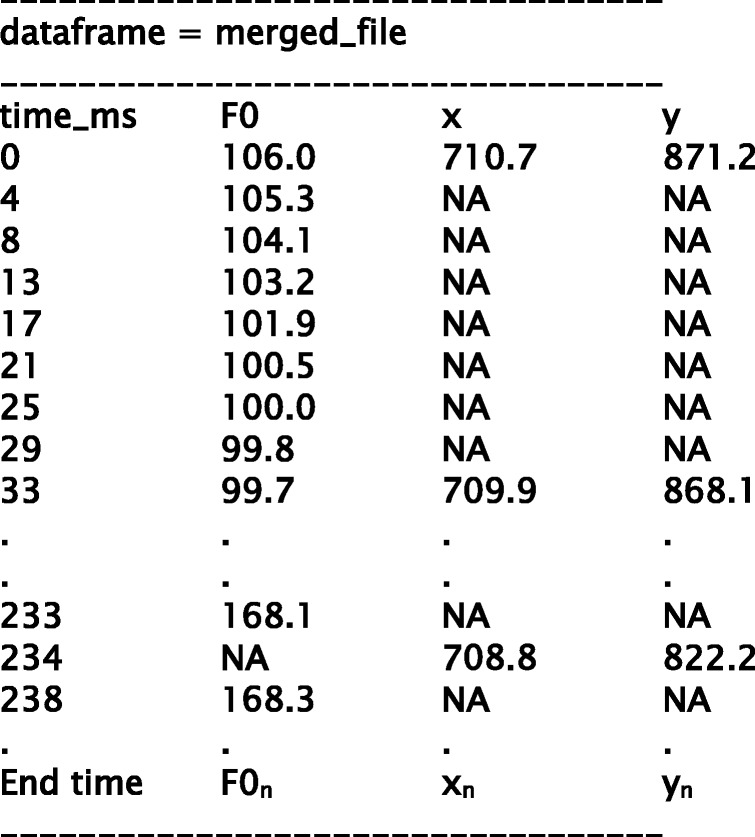



An example of SP_DATA and MOC_DATA merged. Note that from 4 to 29 ms speech is repeatedly sampled, but no *x* and y values are recorded for those times (NAs are given; i.e., “Not Applicable”). Coincidentally, at 33 ms there is an observation for both F0 and motion capture, as the sampling intervals overlapped at that point in time. But at some point the sampling intervals do not align anymore, such that at 234 ms there is an observation for motion capture, but this does not align exactly with the observation 1 ms earlier (233) for F0; thus, two separate rows are constructed in this case.

If both speech and motion tracking are sampled on exactly the same time schedule—that is, identical sampling rate and start time—then the merging function applied above would be the end product for us. However, in the present case there are different sampling rates. Indeed, in most cases there will be some measurement of F0 at time *t* with no complementary motion-tracking data at that time (see Data Example 2). Since we have NAs that are embedded by actual observations for motion tracking and we know the time steps in milliseconds from one known observation to another, we can linearly interpolate the *x* and *y* values for the unknown observations (code can be found in R Code Example 2). We can do this by using the na.approx() function from the R package zoo (Zeileis & Grothendieck, 2005).

**R code Example 2** Code for linear approximation of motion tracking data
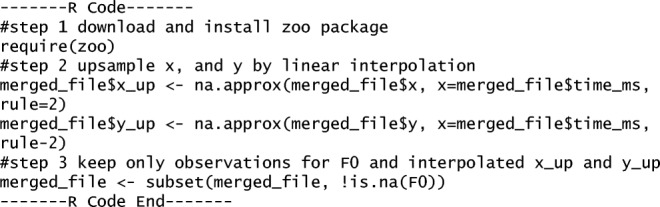


**--------R Code--------**

**#step 1 download and install zoo package**

**require(zoo)**

**#step 2 upsample x, and y by linear interpolation**

**merged_file$x_up <- na.approx(merged_file$x, x=merged_file$time_ms, rule=2)**

**merged_file$y_up <- na.approx(merged_file$y, x=merged_file$time_ms, rule=2)**

**#step 3 keep only observations for F0 and interpolated x_up and y_up**

**merged_file <- subset(merged_file, !is.na(F0) )**

**--------R Code End--------**

“#” indicates a commenting out of the code (the code on this line will not be run by the compiler). This R code firstly loads in the R package zoo (Step 1). Subsequently, it applies the linear interpolation function na.approx two times, which saves two new up sampled variables “x_up” and “y_up” in the original “merged_file” dataframe (Step 2). The na.approx takes as its first argument the variable to be interpolated, the second argument for *x* provides the time index (which is “time_ms”) for the interpolation procedure. The argument type = 2 refers to the procedure that if begin- and endpoints cannot be interpolated (because the begin- and endpoints are not embedded with observations), these values will be extrapolated and given the nearest value. In Step 3, we remove rows that were not of the original sampling rate of F0, effectively keeping all original F0 sampling intervals at a sampling rate of 240 Hz, now also with merged or interpolated *x* and *y* values.

Applying this code from Data Example 2 will give you the updated “merged file” in Data Example 3, whereby *x* and *y* values are up sampled as shown in “*x*_up” and “*y*_up” (through linear interpolation^4^) as to accommodate the sampling rate of speech data (240 Hz).

**Data Example 3** Fully merged data with linearly interpolated motion-tracking data

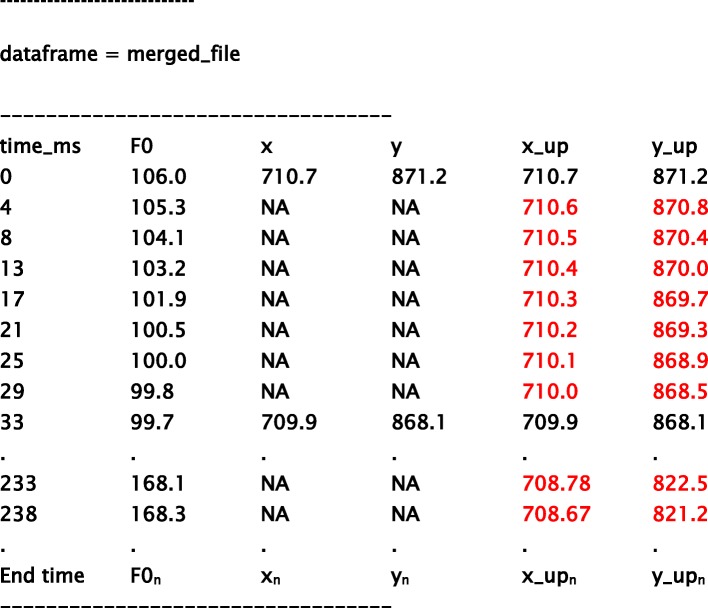



This data example shows the results of up-sampling “x” and “y” into “x_up” and “y_up” using linear interpolation, so as to accommodate the sampling rate of F0. Values shown in red are interpolated values.

Now that we have merged the speech data and with the up-sampled motion-tracking data, we still need to isolate speech + motion-tracking time series for particular events of interest. For example, if we want to know the moment at which gesture *A* reaches its highest vertical point (positive peak *y*), we want to evaluate a subset of time-series values that map onto gesture *A*. Thus we have to merge (ELAN) annotations that marked temporal regions of interest (e.g., gesture type, or gesture identifier event) into the time series. To do this we can let ELAN generate or make a file that has a begin and end time for a particular event tier (gesture event identifier in example below) and we can upload this into R. We thus have our latest “merged_file” within which we want to incorporate the “annotation_data” (see Data Example 4).

**Data Example 4** Annotation file (annotation_data) to be merged with the speech- + motion-tracking data (merged_file)

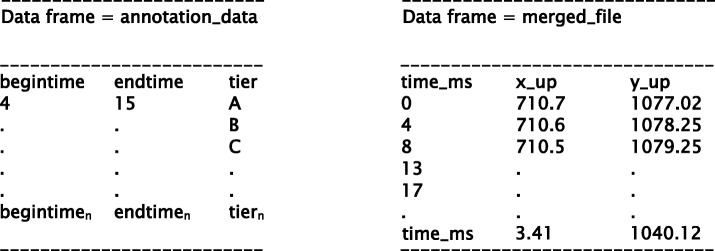



The “annotation_data” file shows a hypothetical gesture event “A” occurring between 4 and 15 ms. We want to place these annotations into our merged speech and motion tracking data, such that for every gesture–speech observation that occurred during an event *x* we will have a variable that marks that observation as belonging to event *x*.

**R Code Example 3** Loading annotation data into time series
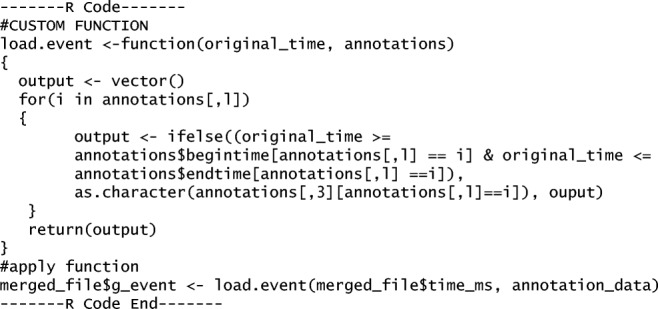


**--------R Code--------**

#CUSTOM FUNCTION

load.event <- function(original_time, annotations)

{

output <- vector()

for(i in annotations[,1])

{

output <- ifelse((original_time >=

annotations$begintime[annotations[,1] == i] & original_time <=

annotations$endtime[annotations[,1] == i]),

as.character(annotations[,3][annotations[,1]==i]), output)

}

return(output)

}

#apply function

merged_file$g_event <- load.event(merged_file$time_ms, annotation_data)

**--------R Code End--------**

This R code constructs a custom-made function that assesses, for an “original_time” vector, which of those values are occurring during some event as given in the “annotations” vector. Specifically, this function loops over the rows of the annotation data and loads an event marker, indicating whether or not an event was happening, into a new vector (called “output”). This is done for each row of the original time series (“original_time”). The final line of code applies this function by entering in the relevant arguments, namely the time_ms vectors of the “merged_file data” and the “annotation_data.”

Applying our custom-made R function (R Code Example 3) will give us a new variable in the “merged_file” called “g_event” (for **g**esture **event**) that marks, for each observation in the time series, whether at that time an event was occurring, on the basis of the annotation begin and end times, which is indicated by an “A” at that time point.

**Data Example 5** The final merged speech and mocap data file, now with annotations

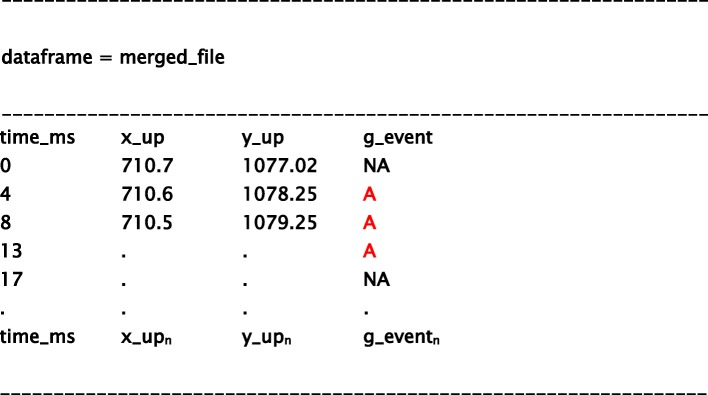



Applying R Code Example 3 produces a new variable, called (“g_event”), that represents that during the observations at “time_ms” 4, 8, and 13, a hypothetical event A (highlighted in red) was occurring. Where NA is given, no “g_event” was occurring.

This final Data Example 5 is a very workable end version of the data. A host of functions can now be applied that take some time series for a given event and compute some measure from it. For example, we can fill a vector “peaks_y” in which for each of the indices we want a maximum vertical position observed during a gesture event (R Code Example 4).

**R Code Example 4** Generating a vector with maximum vertical peaks for each gesture event
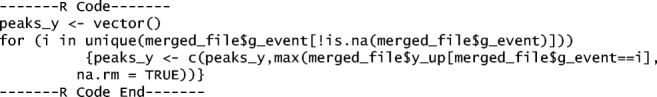


**--------R Code--------**

peaks_y <- vector()

for (i in unique(merged_file$g_event[!is.na(merged_file$g_event)]))

{peaks_y <- c(peaks_y, max(merged_file$y_up[merged_file$g_event==i], na.rm = TRUE))}

**--------R Code End------**

This function extracts all maximum vertical (y_up) positions observed for each g_event (excluding NAs) and orders these values in a vector called peaks_y (from the first observed to the last observed g_event). If we take the mean of the “peaks_y” vector, we would have the average maximum height for all gestures observed.

**Fig. 1:**
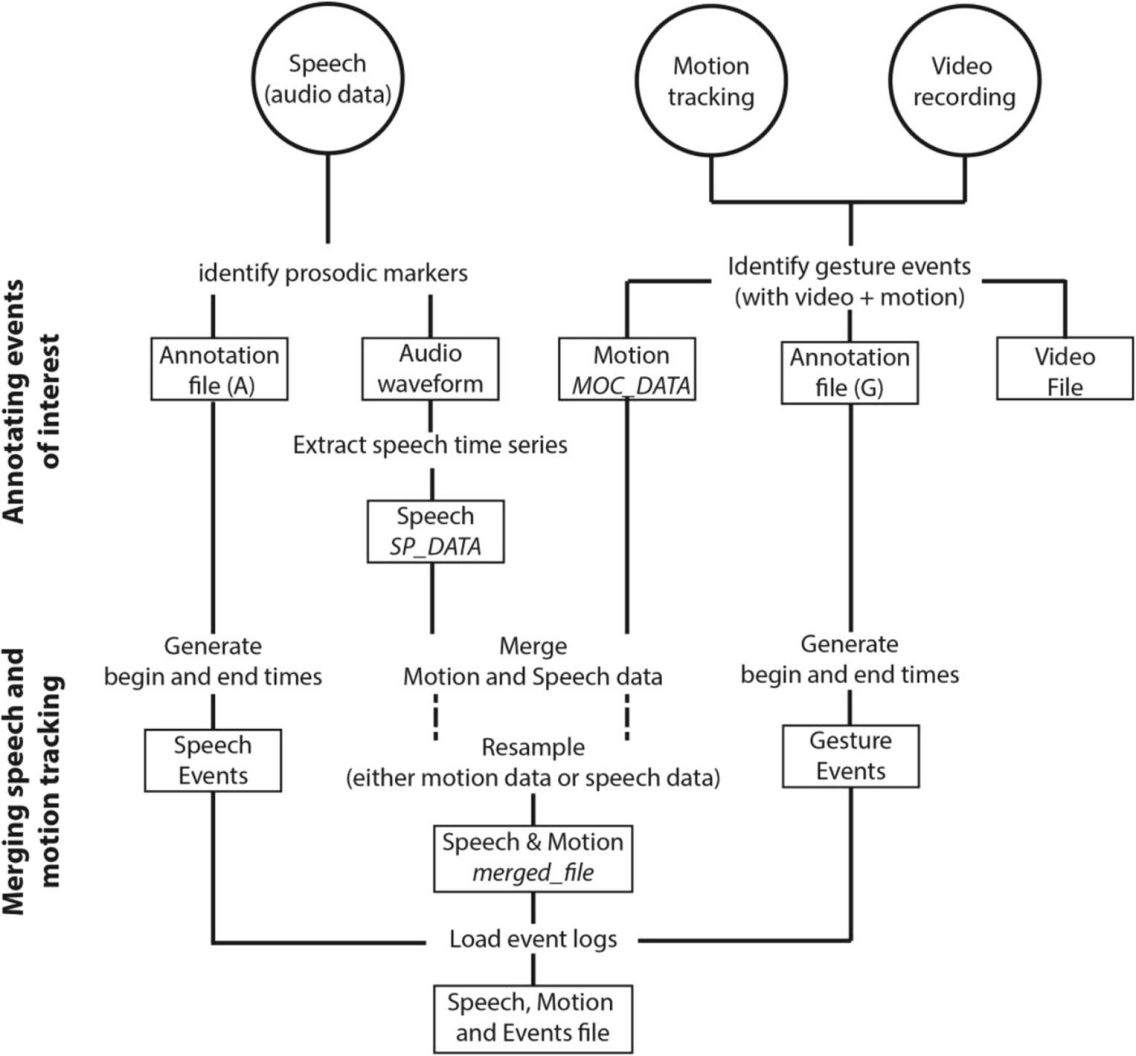
Schematic overview of post-processing steps

A summary of the research pipeline we have presented in Part I can be found in Fig. 1.

## Part II: Comparing performances in temporal estimation of gesture kinematics

In Part I, we provided an overview of the general pipeline of decisions and methodological steps toward quantifying gesture and speech, so that their relationship can be studied. In Part II, we will provide an example of using such methods to measure gesture–speech synchrony, and in doing so, we also provide an assessment of the quality of two video-based motion-tracking methods as compared to the gold-standard wired tracking method. We choose to limit our comparison to these three methods to assess whether such video-based tracking is of sufficient precision to take advantage of the wealth of video-based data already available. The Polhemus system provides an established standard of quality against which to compare the video-based methods.

### Validation analyses

#### Dataset

The present dataset is from an exploratory study by Pouw and Dixon (see, for a preliminary preprint report, Pouw & Dixon, 2018), wherein participants retold a 5-min cartoon that they had just watched (a classic gesture-induction method; McNeill, 1992). During the retelling of the cartoon, the participant’s index finger of the dominant hand was motion-tracked with a Polhemus Liberty (240 Hz; ~ 0.13-mm spatial resolution under ideal conditions). We also recorded speech with a cardioid microphone (RT20 Audio Technica Cardioid microphone) and made video recordings (Sony Digital HD Camera HDR-XR5504), to allow for gesture categorization. In the present study, we used these video recordings to additionally track motion with videography methods. This dataset consists of 392 gesture events. We compare the tracking results from our markered gold standard, Polhemus Liberty, to two video-based methods: deep learning and pixel differencing.

#### Speech acoustics

We extracted the fundamental frequency from the speech data (i.e., pitch track time series) at 240 Hz, with a pitch range = 75–300 Hz. These lower and upper bounds were adjusted for the male voice range of around 85–155 Hz (there were only males in this sample).

### Videography methods

#### Pixel change

Instantaneous pixel change is a quantification of the amount of visual change in the video data, and has been found to be a reliable estimation of gross-body movement that sometimes matches low-cost motion tracking equipment (e.g., Kinect) and even more expensive motion-tracking technology (Romero et al., 2017). We computed the instantaneous pixel change on the video data (sampling rate = NTSC standard sampling rate camera = 29.97 frames per second) using a Python script recently developed and made available by Brookshire and colleagues (2017), for code see github link: https://github.com/gbrookshire/ivc. We applied a low-pass second-order Butterworth filter using R package “signal” (Ligges et al., 2015) of 10 Hz to the pixel change time series. Given that we want to maintain the resolution of speech acoustics (240 Hz) to make a fair comparison to Polhemus, we up-sampled the pixel change time series to 240 Hz.

#### Deep-learning motion tracking (“Deeplabcut”); Minimal versus highly trained network

We trained a pretrained deep neural network (DNN) called “ResNet” with 50 layers (He, Zhang, Ren, & Sun, 2016) for pose estimation for 250,000 iterations. More than 200,000 iterations is a typical amount needed until learning gains plateau as stated by Mathis and colleagues (2018). This DNN yielded 1.73 average pixel difference for the training set, and 2.64 pixel average difference for the test set between human-made estimation of the right hand index finger, versus the estimation made by the DNN (note test pictures were 800 × 1,000 = 8,000 pixels). A full example clip of the DNN motion tracking can be seen at https://osf.io/9hku8/. For a tutorial on DeepLabCut and code, see the github link from Mathis and colleagues: https://github.com/AlexEMG/DeepLabCut.

#### Polhemus video synchronization

For the present purposes, accurate synchronization of audio and motion tracking with the video was of utmost importance for our validation study, because we had to compare videography motion-tracking methods (deep learning and pixel change) with the Polhemus. If video were not aligned with the Polhemus, we could not estimate with certainty how video-based methods perform in comparison to the Polhemus. We performed synchronization of the video and Polhemus data by using the audio-waveform alignment procedure in Part I using Adobe Premiere Pro 2015CC. As such, the video and Polhemus data were completely aligned.

### Temporal estimation of kinematic peak and acoustic peak

**Fig. 2:**
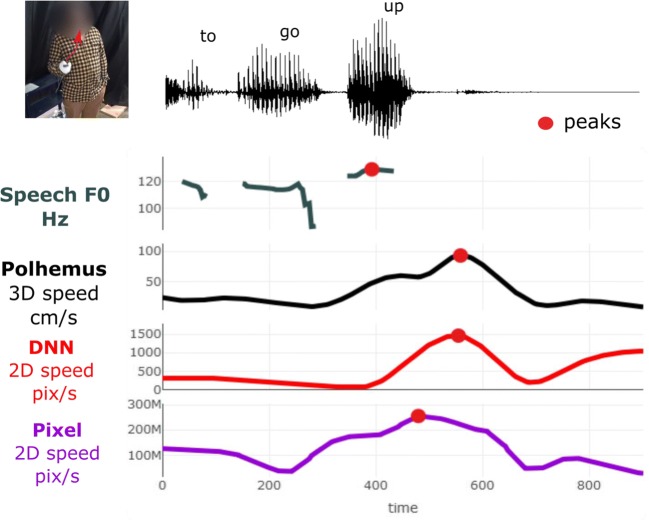
Example gesture event peak speed per method. Example of a gesture event lasting 800 ms (see the video here: https://osf.io/aj2uk/) from the dataset (Event 10 from Participant 2). Red dots indicate the positive maxima peaks in the respective data streams: fundamental frequency in hertz (F0), Polhemus speed in centimeters per second, DNN speed in pixel position change per second, and pixel-method speed in summed pixel change per second. Note that *velocity* is directional speed, whereas *speed* is non-direction-specific velocity

For the present analyses, we wanted to know whether using videography methods to estimate the timing of some kinematic event in gesture, relative to peak F0, is comparable in performance to 3-D high-resolution motion tracking. To make this comparison, we determined for each gesture event when the highest peak in pitch occurred (as an anchor point for speech),^5^ and when the highest peak in speed was observed (peak speed) as determined by the Polhemus, pixel change, and deep neural network methods (see Fig. 2).

### Results

#### Gesture–speech synchrony estimates: Peak speed-peak F0

**Fig. 3:**
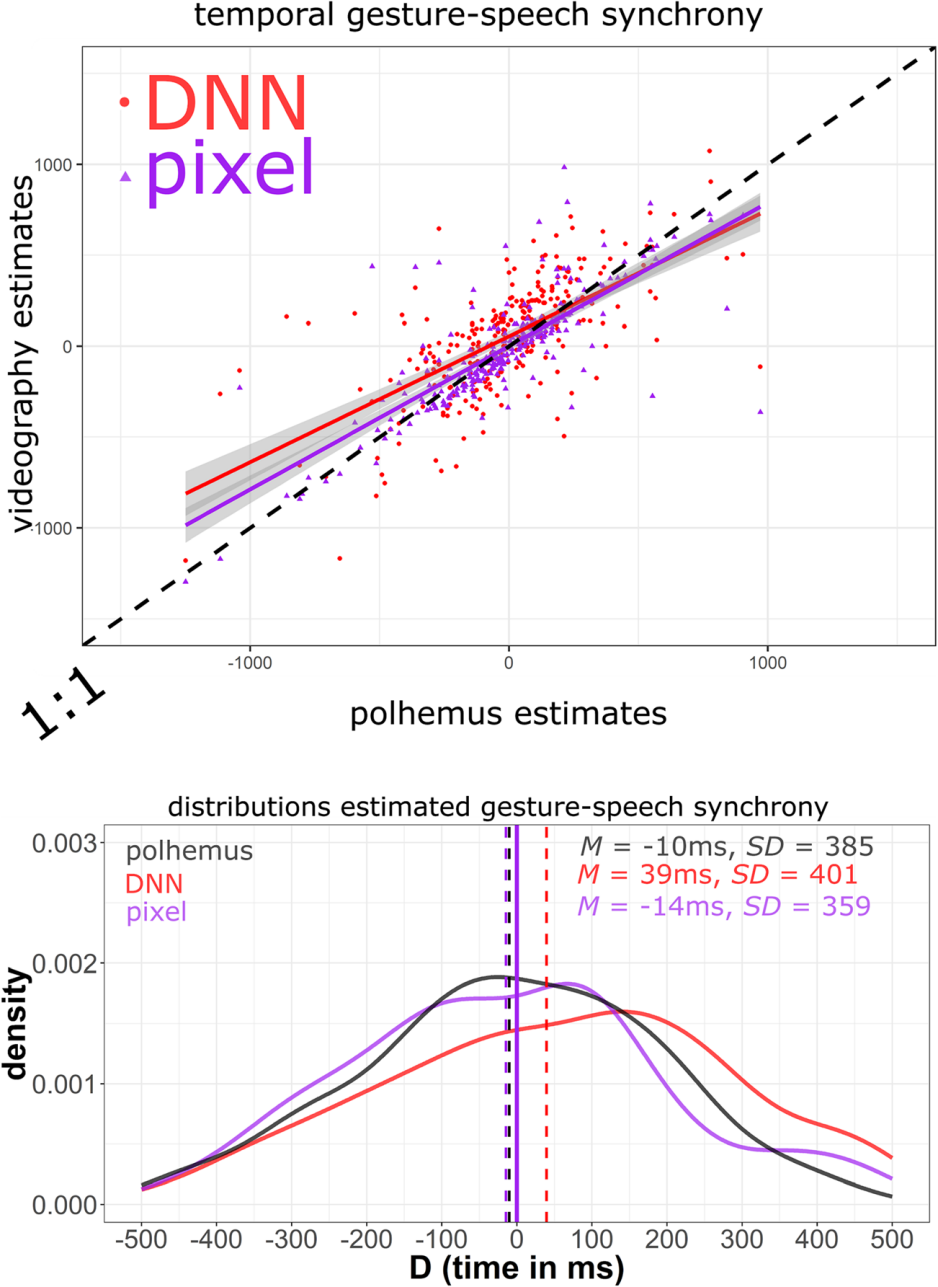
Results and comparisons: Estimated peak speed versus peak F0 in gestures. Upper panel: Videography estimates of gesture–speech synchrony (vertical axis) are compared to Polhemus estimates of synchrony. Purple dots indicate pixel change method performance relative to Polhemus, and red dots indicate deep neural network (DNN) performance relative to Polhemus. A 1:1 slope (as indicated by the black dashed line of identity) would indicate identical performance of videography and Polhemus. Dots along the region of the identity line indicate comparable approximations of gesture–speech synchrony for the different methods. Note that some points are excluded that fell far from the point cloud (for the full graph, go to https://osf.io/u9yc2/). Lower panel: Smoothed density distributions for the estimated gesture–speech synchrony estimates per method, with means (dashed vertical lines) indicating average gesture–speech synchrony

**Fig. 4:**
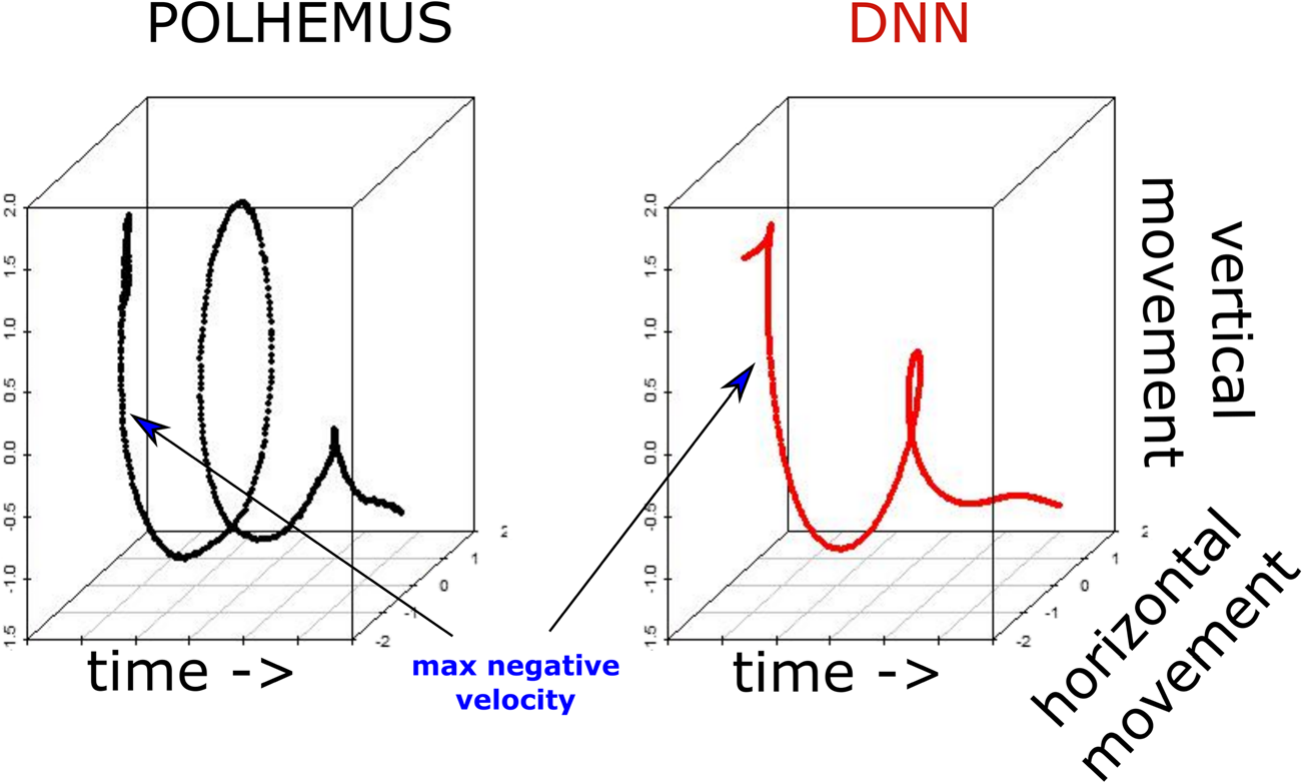
Example trajectory as measured by the Polhemus versus the deep neural network (DNN): Example of an iconic gesture with a circling motion (axis *z*-scaled), as registered by the Polhemus and the DNN. This type of positional information is not available when using the pixel change method. For our comparison, we looked at the moment at which a negative velocity was highest—that is, where a gesture reached its highest speed when moving downward

Table 2 and Figs. 3 and 4, provide an overview of the performance of the videography methods relative to the Polhemus. We observed that both pixel change and the DNN performed well (*r* > .75), as compared to the Polhemus, in estimating gesture–speech synchrony. Given the largely overlapping confidence intervals between DNN and pixel change performance (as compared to Polhemus), we can conclude that both methods are comparable in their performance.

**Table 2:** Results and comparisons estimation peak speed versus peak F0 in gesture

	Polhemus	DNN	Pixel
Estimated mean *(SD)* asynchrony	– 10 ms *(385)*	39 ms *(401)*	– 14 ms *(359)*
Correlation Polhemus			
*r*		.756	.797
95%CI		[.700–.803]	[.750–.837]
*p*		< .00001	< .00001

Note further that the pixel change and DNN methods showed strong correlations (*r*s > .73) in their estimates of the temporal offsets of peak speed and peak F0. The correlation between the two videography methods (DNN and pixel change) was *r* = .747, *p* < .0001.

#### Positional data comparison

The key advantage of DNN motion tracking over the pixel method is that, over and above the quantification of movement, positional information is provided as well. With DNN we could, for example, explore movement trajectories (e.g., Shattuck-Hufnagel & Ren, 2018) for gestures that make some kind of rotational movement (Fig. 4). Or, we could be interested in the maximum vertical points of gestures (e.g., Trujillo et al., 2019). To assess DNN versus Polhemus performance, we instead estimated for each gesture the moment at which the maximum downward speed (or maximum negative speed) was reached, as a way to probe a moment at which a physical impact (or a beat) of a gesture might be produced. This analysis yielded a correlation of the performance of Polhemus versus DNN of *r =* .754, 95% CI [.697, .801], *t*(270) = 18.85, *p* < .0001.

### Discussion

In the first part of the article, we have provided a methodological overview of common challenges in multimodal language research. Our further goal was to make explicit the issues one needs to consider before running an actual experiment, and provide a basic tutorial of some procedures in the post-processing phase. In the second part, we have assessed performance of videography methods, including deep-learning motion tracking, with a common standard motion tracking system (Polhemus Liberty). Specifically, for purposes of estimating gesture–speech synchrony, we showed that both pixel change methods and deep neural network motion tracking are performing very well relative to a Polhemus Liberty wired motion-tracking system. Deep-learning motion tracking has the further advantage of being able to track the 2-D position of the gesture, rather than only a quantification of the amount of movement, as is the case for pixel change methods.

Although performance of the deep-learning motion tracking was high in our study, some parameters may need to be adjusted in order to make this technique more reliable for future studies. For example, performance might be enhanced by using a larger training dataset, providing more accurate position judgments of hand positions from a second independent coder, or by interpolating the hand position when DeepLabCut indicates low certainty during tracking. However, for present purposes, we show that DeepLabCut performs very well in our task of estimating gesture–speech synchrony.

We think this present validation of video-based tracking is important because it shows that reliable motion tracking for gesture–speech synchrony analyses can be done without the need to collect additional data. Of course, physical motion tracking systems, whether optical or electromagnetic, will remain superior to video-based tracking that relies on a single point of view. However, given the present high performance of the videography methods, we think such methods promises to be a major step forward in terms of efficiency and reliability of tracking meaningful movements.

#### Implications and applications

We hope that the present article contributes to the study of multimodal language in more diverse populations and with increasingly larger samples to accommodate the study of individual differences. To serve these goals, the steps we have described here can be applied to any type of motion-tracking data that can be reliably synchronized with audio/video data. Multimodal language researchers can apply these quantitative methods to data that has already been acquired, or they can choose to take motion tracking requirements into account when collecting new data. The use of already acquired data is particularly useful given the large number of video corpora that have been generated and maintained over the years. Additionally, markerless motion tracking (whether video-based or device-based) can be quite valuable for capturing movements of more sensitive populations (e.g., Eigsti & Pouw, 2018; Romero et al., 2018). Finally, although we have assessed deep-learning motion-tracking performance in terms of the temporal estimation of kinematic peaks, this method can be especially useful for gesture trajectory analyses (e.g., Shattuck-Hufnagel & Ren, 2018), and are likely to replace methods that require annotations by hand (e.g., Hilliard & Cook, 2017).

#### Summary

The temporal relationship between speech and gesture is an integral part of the study of multimodal language. Although methods are now available for objectively quantifying both movement and speech, bringing these two streams of data together for meaningful and reliable analyses is nontrivial. We have provided an overview of the key steps that must be taken in order to conduct such research, and have described different approaches that are available at each step. The examples and code provided in the present article should enable multimodal researchers to perform quantitative analyses of gesture–speech coordination (for a guided video tutorial see also https://osf.io/rxb8j/). Finally, we validated the cheap, video-based motion-tracking techniques for quantifying speech–gesture synchrony. We hope that this overview will provide a useful resource for multimodal language researchers interested in applying quantitative methods to the study of speech and gesture.

## Footnotes

1 Note that some video cameras allow for an audio plugin, which automatically synchronizes the specialized audio with the video stream. This method can be particularly useful when tracking of motion is performed on the video data alone (because no post-hoc audiovisual motion synchronization is needed anymore).

2 Note that previous Adobe Premiere versions may not support this function. For example, Adobe Premiere Pro Cs6 does not have an audio synchronization function.

3 What if a researcher wants to align two separately recorded cardioid microphone streams with different contents (such that not enough information in the audio waveforms of the microphones overlap)? A chaining solution would be to use the in-built audio of a camera (which does capture both audio streams), and sync both microphone streams to the camera, rather than directly to each other (leading to synchronized microphone data). There are other solutions to this problem, as well, but the chaining technique is quite useful for post-hoc synchronization.

4 Other interpolation methods are possible as well, such as cubic spline interpolation.

5 It is open for discussion whether a peak in F0 is a good anchor point (rather than, say, peak amplitude or ToBi prosody-label-based point estimates). However, this is not something that should be of present concern. As long as we have a formally defined anchor point in speech, we can do the planned comparisons of the motion-tracking methods in terms of gesture–speech synchrony estimations.
